# Aminopropyltransferases Involved in Polyamine Biosynthesis Localize Preferentially in the Nucleus of Plant Cells

**DOI:** 10.1371/journal.pone.0046907

**Published:** 2012-10-08

**Authors:** Borja Belda-Palazón, Leticia Ruiz, Esmeralda Martí, Susana Tárraga, Antonio F. Tiburcio, Francisco Culiáñez, Rosa Farràs, Pedro Carrasco, Alejandro Ferrando

**Affiliations:** 1 Instituto de Biología Molecular y Celular de Plantas CSIC-Universidad Politécnica de Valencia, Valencia, Spain; 2 Instituto Andaluz de Investigación y Formación Agraria, Centro La Mojonera, Almería, Spain; 3 Unidad de Fisiología Vegetal, Universidad de Barcelona, Barcelona, Spain; 4 Centro de Investigación Príncipe Felipe, Valencia, Spain; 5 Departamento de Bioquímica y Biología Molecular, Universidad de Valencia, Valencia, Spain; Lawrence Berkeley National Laboratory, United States of America

## Abstract

Plant aminopropyltransferases consist of a group of enzymes that transfer aminopropyl groups derived from decarboxylated S-adenosyl-methionine (dcAdoMet or dcSAM) to propylamine acceptors to produce polyamines, ubiquitous metabolites with positive charge at physiological pH. Spermidine synthase (SPDS) uses putrescine as amino acceptor to form spermidine, whereas spermine synthase (SPMS) and thermospermine synthase (TSPMS) use spermidine as acceptor to synthesize the isomers spermine and thermospermine respectively. In previous work it was shown that both SPDS1 and SPDS2 can physically interact with SPMS although no data concerning the subcellular localization was reported. Here we study the subcellular localization of these enzymes and their protein dimer complexes with gateway-based Bimolecular Fluorescence Complementation (BiFC) binary vectors. In addition, we have characterized the molecular weight of the enzyme complexes by gel filtration chromatography with *in vitro* assembled recombinant enzymes and with endogenous plant protein extracts. Our data suggest that aminopropyltransferases display a dual subcellular localization both in the cytosol and nuclear enriched fractions, and they assemble preferably as dimers. The BiFC transient expression data suggest that aminopropyltransferase heterodimer complexes take place preferentially inside the nucleus.

## Introduction

Polyamines are small aliphatic polycations present in all eukaryotes, and in flowering plants the most abundant are the diamine putrescine, the triamine spermidine and the tetraamines spermine and thermospermine, each of them with specific biological functions [1]. According to the relevant physiological roles allocated to polyamines one would expect a stringent control of homeostasis, and indeed these compounds are subjected to strict metabolic control by means of elaborated anabolism [2], catabolism [3] and conjugation pathways [4], [5], [6]. The polyamine biosynthesis pathway in plants has received most of the initial attention in the field, taking advantage of conserved pathways in other eukaryotic organisms and additional enzymes incorporated by the cyanobacterial ancestor of the chloroplast. Two alternate routes for putrescine biosynthesis are therefore present in plants: (i) the unique among eukaryotes arginine decarboxylation pathway located mainly in chloroplasts, and (ii) the ornithine decarboxylation pathway, present also in the rest of eukaryotes, which is mainly found in the cytosol [2]. Strikingly the ornithine pathway has lost most of its regulatory components in plants and it is even totally absent in *Arabidopsis thaliana* [7]. The arginine pathway for putrescine biosynthesis consists of three enzymes acting sequentially, namely arginine decarboxylase (ADC; EC 4.1.1.19), agmatine deiminase/iminohydrolase (AIH; EC 3.5.3.12), and *N*-carbamoylputrescine amidohydrolase/amidase (NCPAH; EC 3.5.1.53). After putrescine synthesis, next biosynthetic steps require the activity of *S*-adenosylmethionine decarboxylases (SAMDC; EC 4.1.1.50) to provide dcSAM. Putrescine, then, serves as the acceptor for the dcSAM-dependent transfer of aminopropyl groups catalyzed by the aminopropytransferases spermidine synthases (SPDS; EC 2.5.1.16) to produce spermidine. In *Arabidopsis* two genes: *SPDS1* (At1g23820) and *SPDS2* (At1g70310) code for SPDS activity [8] and four genes *SAMDC1-4* (At3g02470, At3g25570, At5g15959, At5g18930) code for SAMDC [9]. The last enzymatic step of polyamine biosynthesis catalyzes the dcSAM-dependent transfer of aminopropyl groups to propylamine acceptor spermidine, to produce either spermine by the action of spermine synthase (SPMS; EC 2.5.1.22) or its natural isomer thermospermine, by the activity of thermospermine synthase (TSPMS; EC 2.5.1.79). In *Arabidopsi*s these aminopropyltransferase enzymatic activities are encoded by single genes, namely *SPMS* (At5g53120) for spermine synthase [8], and *ACL5* (At5g19530) for thermospermine synthase [10]. In terms of evolution, it seems that all spermidine synthases evolved from a common ancestor prior to the separation between prokaryotes and eukaryotes, giving rise later to novel activities: on the one hand independent origins of SPMS in animals, fungi and angiosperm plants, and on the other hand a change in activity to TSPMS in both archaea and bacteria that was later horizontally transferred to plants [11]. Curiously, the independently evolved *SPMS* gene in angiosperms clusters closer to spermidine synthases than its functional metazoan orthologs and far from the *ACL5* gene encoding TSPMS active enzyme. These evolutionary features correlate with functional data of multiprotein complex assembly, since protein-protein interactions between aminopropyltransferases have been described in *Arabidopsis* for SPDS1, SPDS2 and SPMS, but not for TSPMS [8]. In spite of vast amount of information with regard to plant aminopropyltransferases [12], one relevant question that remains unanswered is related to the subcellular localization of the individual enzymes and the enzymatic complexes. Here we explore the subcellular localization of aminopropyltransferase enzymes by immunohistochemistry and with the use of translational fusions to the green fluorescence protein (GFP), and we also study the localization of enzyme complexes by means of gateway-based binary vectors that allow Bimolecular Fluorescence Complementation (BiFC) studies in planta. Estimation of molecular weights by gel filtration chromatography support the formation of both homo and heterodimeric enzyme complexes. From these studies we conclude that *Arabidopsis* aminopropyltransferases show a dual cytosol/nuclear localization, and the heterodimer complexes localize preferentially within the nucleus.

## Materials and Methods

### Plant Material

*Arabidopsis* wild type (Col-0) plants were grown in pots on a mix of 25% perlite, 25% vermiculite and 50% soil, for two to three weeks in environmental growth chamber under long-day photoperiod cycles (16 hours light at 21°C and 8 hours dark at 19°C) with a light intensity of 110 µmol m^−2^ s^−1^. *Arabidopsis* cell line T87 was cultured as previously described [13]. *Nicotiana benthamiana* seeds were sown on a mix of 50% vermiculite and 50% soil and grown for three to four weeks in controlled greenhouse conditions under long-day photoperiod cycles (16 hours light/8 hours dark) at 22°C±1°C.

### Design of BiFC Vectors and Cloning Procedures for BiFC and GFP Translational Fusion Constructs in Binary Plasmids

To create the gateway destination vectors pYFN43 and pYFC43, coding sequences of the YFP encoding gene were PCR amplified from plasmids pBiFC-YN155 and pBiFC-YC155 [14] using the following primer pairs:

5′-GGGGTACC **ATGGTGAGCAAGGGCGAGGAGCTGTT-**3′ and

5′-GGGGCGCGCC *AAGAGATCCACCTCCACCAGATCCACCTCCACCAGATCCACCTCCACCGGC* **CATGATATAGACGTTGTGGCTGTTGTAGTT**-3′ to amplify the N-terminal sequence of YFP corresponding to residues 1 to 154 including a flexible linker shown in italics, underlined cloning sites *Kpn* I and *Asc* I and coding sequence in bold. The C-terminal sequence of YFP corresponding to residues 154 to 240 was PCR amplified using the primer pairs:

5′-GGGGTACCATGGCCGACAAGCAGAAGAACGGCAT-3′ and

5′-GGGGCGCGCC *AAGAGATCCACCTCCACCAGATCCACCTCCACCAGATCCACCTCCACCGGC* **CTTGTACAGCTCGTCCATGCCGAGAGTGAT**-3′ with the flexible linker in italics, underlined cloning sites *Kpn* I and *Asc* I and coding sequence in bold. Both PCR products were cloned after *Kpn* I-*Asc* I restriction into plasmid pMDC43 [15] after removing the GFP6 coding sequence with *Kpn* I and *Asc* I to generate the plasmids pYFN4 and pYFC43. Both plasmids contain *attR* sites that allow the direct cloning by recombination to yield ‘in frame’ fusion of appropriate coding sequence with *attL* flanking sites to either the N-terminal fragment of the YFP protein (residues 1–154) in pYFN43, or to the C-terminal fragment of the YFP protein (residues 154–240) in pYFC43. To enhance folding of the fusion proteins a flexible linker was introduced between the coding sequence of either fragment of YFP and the *attR* recombination site. Sequences can be retrieved and plasmids can be requested from the web page http://www.ibmcp.upv.es/FerrandoLabVectors.php. All clones were introduced into destination vectors pYFN43 and pYFC43 by LR gateway-based recombination with entry clones. Positive colonies were selected with 50 µg/mL amikacin (Sigma, Saint Louis, Missouri, USA). Entry clones containing the protein-protein interaction coding sequences from *AKIN10* and *AKINβ2* were obtained as follows. *AKINβ2* partial coding sequence was subcloned from a previous construct in pPE1000 [13] as a *Nco* I-*Bgl* II (blunt) fragment into pENTR11(Invitrogen, Life Technologies, Grand Island, NY, USA) digested with *Nco* I-*Eco* RV. AKIN10 coding sequence from a pGEM vector [13] was subcloned as *Eco* RI-*Sal* I (blunt) fragment into pENTR3C (Invitrogen, Life Technologies, Grand Island, NY, USA) *Eco* RI-*Eco* RV digested.

Aminopropyltransferase coding sequences without stop codon were cloned as follows. cDNA synthesized from total RNA isolated from *Arabidopsis* wild type plants was used as a template to amplify by PCR coding sequences with the following primer pairs for *SPDS1*:

5′-GG*ACAAGTTTGTACAAAAAAGCAGGCT*TA**ATGGACGCTAAAGAAACCTCT-**3′ and

5′-GG*ACCACTTTGTACAAGAAAGCTGGGT*C**ATTGGCTTTTGACTCAAT-**3′.

The *attB* recombination sites are indicated in italics and the coding sequences in bold. In a similar way the following primer pairs were used for *SPDS2*:

5′-GG*ACAAGTTTGTACAAAAAAGCAGGCT*TA**ATGTCTTCAACACAAGAAGCG** **-**3′ and

5′-GG*ACCACTTTGTACAAGAAAGCTGGGT*C**GTTGGCTTTCGAATCAAT-**3′.

The same strategy was followed for cloning *SPMS* coding sequence with the following primer pairs:

5′-GG*ACAAGTTTGTACAAAAAAGCAGGCT*TA**ATGGAGGGAGACGTCGGAATA**-3′ and

5′-GG*ACCACTTTGTACAAGAAAGCTGGGT*C**AGAAGCCAGAAGTGAAGC-**3′.

The purified PCR products were used for BP gateway-based recombination reaction with pDONR-Zeo (Invitrogen, Life Technologies, Grand Island, NY, USA) to obtain the entry clones for each aminopropyltransferase that could be used for a subsequent LR gateway-based recombination reaction with either pYFN43 and pYFC43 for BiFC assays, pMDC43 [15] for GFP N-terminal translational fusion, pMDC83 [15] for GFP C-terminal translational fusion and pGWB455 [16] for mRFP N-terminal translational fusion. The binary constructs thus obtained were introduced into *Agrobacterium tumefaciens* GV3101 pMP90 as described [13].

### Plant Transformation and BiFC Assays

*N. benthamiana* leaves were transformed by injection of *A. tumefaciens* GV3101/pMP90 cells harbouring the appropriate plasmids as follows. To suppress gene silencing, *A. tumefaciens* cells expressing the p19 protein of the tomato bushy stunt virus [17], from Plant Bioscience Limited (PBL, Norwich, UK), were used in the co-infiltration procedure. Overnight grown cultures of *A. tumefaciens* of about 2.0 OD_600_ units were collected and resuspended in similar volume of infiltration buffer (MgCl_2_ 10 mM, MES 10 mM pH 5.6, acetosyringone 200 µM) and incubated in a rocking platform (Kuhner, Basel, Switzerland) at 28°C for 3 to 4 hours. A mixture of *Agrobacterium* strains containing the fluorescent translational fusion constructs and the p19 plasmid at OD_600_ 1.0∶1.0∶1.0 was prepared for co-infiltration into the abaxial air space of *N. benthamiana* leaves with a needleless syringe. Epidermal cell layers of at least two transformed leaves of 3–4 plants of similar age were assayed for fluorescence under confocal microscope 3–4 days after infiltration. The experiments were repeated at least 3 times for every construct.

*Arabidopis* wild type plants were stably transformed with constructs in pMDC83 binary vector according to the floral dip protocol [18], and hygromycin resistant T1 transgenic plants were selected in MS agar plates with antibiotic. An average of 15 to 20 T1 transgenic seedlings were obtained for each transformation and at least 4 were used for selecting T2 transgenic plants resistant to hygromycin with a 3∶1 ratio. An average of 10 plants from the 4 independent T2 lines for each construct were used for direct visualization of GFP fluorescence with confocal microscopy, and the required amount of T2 seedlings was used for the nuclear fractionation studies.

### Recombinant Protein Expression and Antibody Production

Aminopropyltransferase coding sequences without stop codon were cloned as follows. cDNA synthesized from total RNA isolated from *Arabidopsis* wild type seedlings was used as a template to amplify by two-step sequential PCR the aminopropyltransferase coding sequences including a Protease 3C cleavage site (GE Healthcare, UK). The following primer pairs were used for *SPDS1* in the first PCR round:

5′-CTGTTCCAGGGGCCC **ATGGACGCTAAAGAAACCTCT** *-*3′ and

5′-GG*ACCACTTTGTACAAGAAAGCTGGGTC* **ATTGGCTTTTGACTCAAT**-3′.

The following primer pairs were used for *SPDS1* in the second PCR round:

5′-GG*ACAAGTTTGTACAAAAAAGCAGGCT*TACTGGAAGTTCTGTTCCAGGGG CCC **ATG**-3′ and

5′-GG*ACCACTTTGTACAAGAAAGCTGGGTC* **ATTGGCTTTTGACTCAAT**-3′.

The *attB* recombination sites are indicated in italics, the Protease 3C cleavage site underlined and the coding sequences in bold.

In a similar way the following primer pairs were used for *SPDS2* in the first PCR round:

5′-CTGTTCCAGGGGCCC **ATGTCTTCAACACAGAAGCG**-3′ and

5′-GG*ACCACTTTGTACAAGAAAGCTGGGTC* **GTTGGCTTTCGAATCAAT**-3′.

The following primer pairs were used for *SPDS2* in the second PCR round:

5′-GG*ACAAGTTTGTACAAAAAAGCAGGCT*TACTGGAAGTTCTGTTCCAGGGG CCC **ATG**-3′ and

5′-GG*ACCACTTTGTACAAGAAAGCTGGGTC* **GTTGGCTTTCGAATCAAT**-3′.

The same strategy was followed for cloning *SPMS* coding sequence with the following primer pairs in the first PCR round:

5′-CTGTTCCAGGGGCCC **ATGGAGGGAGACGTCGGAATA**-3′ and

5′-GG*ACCACTTTGTACAAGAAAGCTGGGTC* **AGAAGCCAGAAGTGAAGC**-3′.

The following primer pairs were used for *SPMS* in the second PCR round:

5′-GG*ACAAGTTTGTACAAAAAAGCAGGCT*TACTGGAAGTTCTGTTCCAGGGG CCC **ATG**-3′ and

5′-GG*ACCACTTTGTACAAGAAAGCTGGGTC* **AGAAGCCAGAAGTGAAGC**-3′.

The purified PCR products were used for BP gateway-based recombination reaction with pDONR-Zeo (Invitrogen, Life Technologies, Grand Island, NY, USA) to obtain the entry clones for each aminopropyltransferase that were used for a subsequent LR gateway-based recombination reaction with pDEST17 (Invitrogen, Life Technologies, Grand Island, NY, USA) to add a His-tag translational fusion. The final clones were introduced into *E. coli* strain BL21-CodonPlus (DE3) (Stratagene-Agilent, Santa Clara, CA, USA) for heterologous protein expression. To purify recombinant His-tag proteins 250 mL of transformed *E. coli* cultures carrying appropriate constructs grown at 37°C to OD_600_ of 0.3–0.4 were induced by adding IPTG 0.8 mM and further grown for 3 hours at 28°C. Collected cells were resuspended with 1 mL protein extraction buffer containing 20 mM Tris-HCl pH 7.6, 300 mM NaCl, 1 mM DTT, 20 mM imidazole and 20 µL of protease inhibitor cocktail (Sigma, Saint Louis, Missouri, USA), sonicated, and centrifuged for 15 minutes to remove cell debris and insoluble material. Total soluble proteins were loaded on a 1 mL His Trap™ HP (GE Healthcare, UK) previously equilibrated with binding buffer (20 mM Tris-HCl pH 7.6, 300 mM NaCl, 20 mM imidazole) at a flow rate of 1 mL/min. Protein elution was achieved through a linear gradient from 20 mM to 500 mM imidazole and 1 mL fractions were collected and used for coomassie staining of SDS-PAGE gels and immunoblot analysis. Monoclonal anti-His antibodies (Novagen, Merck, Darmstadt, Germany) were used at 1∶1000 dilution for western blot.

The recombinant GST-SPDS2 fusion protein isolated as previously described [8] was used to inject rabbits for antibody production. An aliquot of the recombinant GST-SPDS2 protein was cleaved with Factor Xa protease to isolate pure SPDS2 protein. Antibodies specific to SPDS2 were precipitated from crude total immune serum by ammonium sulphate and affinity purified by binding to SPDS2 purified protein blotted on nitrocellulose [19]. Purified antibody was used at 1∶100 dilution for western blot studies whereas crude serum polyclonal antibodies were used at 1∶2500 dilution.

All secondary antibodies were used for immunoblotting at 1∶5000 dilution including anti-rabbit polyclonal antibodies coupled to either peroxidase (Santa Cruz Biotechnology, Santa Cruz, CA, USA) or alkaline phosphatase (Thermo-Pierce, Rockford, IL, USA) as well as anti-mouse monoclonal antibodies conjugated to peroxidase (Santa Cruz Biotechnology, Santa Cruz, CA, USA). Immunoblot detection was achieved using the chemiolumiscent ECL detection kit (GE Healthcare, UK) for peroxidase coupled antibodies or the NBT-BCIP western detection (Thermo-Pierce, Rockford, IL, USA) for secondary antibodies conjugated to alkaline phosphatase.

### Immunohistochemistry and Fluorescence Confocal Microscopy

For immunolocalisation, an all-purpose fixative (80% v/v ethanol, 3.5% v/v formaldehyde, 5% v/v acetic acid) was used for paraffin embedding [20]. Sections from paraffin-embedded material were blocked with 3% goat serum in PBS (10 mM phosphate, 150 mM NaCl, pH 7.4) for 30 min at 22°C and incubated with polyclonal anti-SPDS antibody diluted 1∶500. Immunoreactivity was visualised by the avidin-biotin complex (Vectastain Elite ABC kit; Vector, Burlingame, CA, USA) using diaminobenzidine as substrate for peroxidase. At least 10 biological replicates were used and observed.

Confocal imaging was carried out with a Leica True Confocal Scanning (TCS) laser microscope. Visualization of GFP fluorescence was achieved by sample excitation with Argon laser at 488 nm with a 500 nm beamsplitter and the spectral detection was set between 510 and 535 nm. For mRFP detection, the excitation was performed with Helium-neon laser at 543 nm with a double band dicroic mirror (488/543) and spectral detection between 564 and 610 nm. Image analysis was carried out with Leica confocal software. To prepare agroinfiltrated *N. benthamiana* leaves for confocal imaging, 1–2 cm diameter leaf sections were mounted on a microscope slide and covered with Mowiol® mounting medium prepared according to supplier (Polysciences, Warrington, PA, USA) for observation through the leaf abaxial side.

### Biochemical Fractionation

Nuclear fractionation was performed with slight modification of previously reported protocols [21]. Around 1.5 grams of two weak old *Arabidopsis* seedlings of SPDS2-GFP T2 transgenic lines were ground in lysis buffer (20 mM Tris-HCl pH 7.4, 25% glycerol, 20 mM KCl, 2 mM EDTA, 2.5 mM MgCl_2_, and 250 mM sucrose) containing plant protease inhibitor cocktail (Sigma, Saint Louis, Missouri, USA) and 1 mM phenylmethylsulfonyl fluoride (PMSF). The lysate was filtered through two layers of Miracloth (Calbiochem, Merck, Darmstadt, Germany) and centrifuged at 1,000 g for 10 min to pellet the nuclei. The cytosolic fraction was removed until use and the nuclear pellet was washed 2–4 times in nuclei resuspension buffer (20 mM Tris-HCl pH 7.4, 25% glycerol, 20 mM KCl, and 0,5% Triton X-100). The nuclear pellet was finally resuspended in 0.1 mL of medium salt buffer (20 mM Tris-HCl pH 7.4, 0.4 M NaCl, 1 mM EDTA, 5% glycerol, 1 mM 2-mercaptoethanol, 0.1% Triton X-100, 0.5 mM PMSF, and plant protease inhibitor cocktail (Sigma, Saint Louis, Missouri, USA) and then frozen and thawed and used for western blot analysis. The purity of the different fractions was shown using antibodies against histone H3 (Abcam, Cambridge, MA, USA), and Ponceau staining of the ribulose-1,6-bisphosphate carboxylase.

### Gel Filtration Chromatography

To investigate the behaviour of plant aminopropyltransferases on gel filtration chromatography, about 2 mg of total protein extract derived from *Arabidopsis* T87 plant cell suspension [13] prepared as described [8] was size fractionated either on a HiPrep Sephacryl S300 (16/60, bed volume approximate 120 mL; GE Healthcare, UK) or on a Superose 6 HR (10/30 bed volume approximate 24 mL; GE Healthcare, UK) previously calibrated with molecular mass standards (Sigma MWGF1000). The flow rate was set to 0.5 mL/min in both cases and 1 mL fractions were collected and concentrated to 0.2 mL with Vivaspin (GE Healthcare, UK) and aliquots of 30 µL were directly used for immunoblot analysis with affinity purified anti-SPDS antibody.

Approximately 50 to 100 µg of each affinity purified recombinant His-tagged aminopropyltransferase was loaded onto a Superdex 200 (10/300 GL, bed volume approximate 24 mL; GE Healthcare, UK) with a flow rate of 0.2 mL/min previously equilibrated with buffer containing 20 mM Tris-HCl pH 7.6 and 150 mM NaCl, and 1.5 mL fractions were scored for OD_280_ determination. Image of elution profile was acquired with software Unicorn 5.2 (GE Healthcare, UK).

## Results

### Tissue Distribution of Spermidine Synthases in *Arabidopsis*

**Figure 1:**
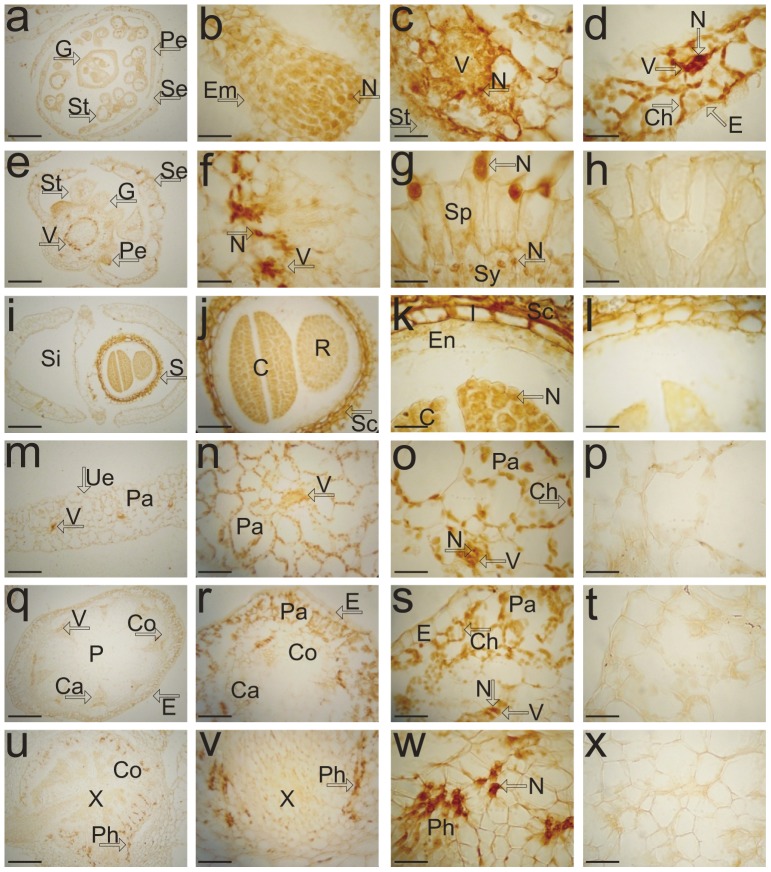
Histological immunolocalisation of SPDS aminopropyltransferases in *Arabidopsis*. Paraffin-embedded sections of different organs of *A. thaliana* were incubated with anti-SPDS antibodies and an avidin-biotin-peroxidase detection system. Dark brown staining indicates SPDS antibody-specific reaction. Either transverse or oblique sections are shown for: (a) wild-type developed flower, (b) embryo sac, (c) stamen, (d) sepal, (e) wild-type flower primordia, (f) receptacle, (g) gynoecium (i) mature silique, (j, k) embryo, (m, n, o) leaf, (q, r, s) stem, and (u, v, w) root. Specificity of the signal is shown by using preimmune serum as control reactions in (h, l, p, t, x). Abbreviations: C, cotyledon; Ca, cambium; Ch, chloroplast; Co, cortex; E, epidermis; Em, embryo sac; En, peripheral endosperm; G, gynoecium; I, inner integument; N, nucleus; P, pith; Pa, palisade mesophyll; Pe, petal; Ph, phloem; R, radicle; S, embryo; Sc, seed coat; Se, sepal; Si, silique; Sp, stigmatic papillae; St, stamen; Sy, style; Ue, upper epidermis; V, vascular bundle; X, xylem. Bar = 100 µm in (a, e, i, m, q, u); 25 µm in (j, n, r, v); 10 µm in (b, c, d, f, g, h, k, l, o, p, s, t, w, x).

To assess the tissue pattern of *Arabidopsis* aminopropyltransferases we have used polyclonal antibodies raised against *Arabidopsis* SPDS2 to perform immunohistochemical localization in *Arabidopsis* plant tissues. Prior to the immunostaining studies we evaluated the biochemical properties of the antibody comparing the cross-reactivity of crude serum versus affinity purified antibody (Figure S1, panel A). Taking into account that the antibody was raised against the recombinant full-length fusion protein GST-SPDS2, and due to large sequence similarity among all three aminopropyltransferases (82.7% between SPDS1 and SPDS2 and around 56% between SPMS and either SPDS1 or SPDS2) we tested the immunogenic properties of the antibody against all three recombinant proteins expressed in *E. coli*. As shown in the panel B of Figure S1, the antibody weakly cross-reacted against SPDS1 compared to SPDS2 and showed no apparent cross-reaction against SMPS. Therefore, the immnunohistochemical results obtained with the crude serum should be interpreted as a combined signal for both SPDS1 and SPDS2 proteins, mostly specific for the latter, with some possible residual background due to GST antigenicity. Figure 1 shows the protein distribution pattern in *Arabidopsis* histological sections with a clear nuclear staining in embryo sac, stigma and style (Figure 1 b, g) and also in the nucleus of phloem tissue in stamens, sepals and receptacle (Figure 1 c, d, f). Weak signal could be also detected in both stamen plastids and sepal chloroplasts (Figure 1 c, d). Within the seed, the embryo cotyledons and radicle displayed a difused immunostaining signal between nucleus and cytoplasm (Figure 1 k). The immunostaining pattern in vegetative tissues showed similar subcellular localization with the most intense signals in the nucleus of phloem tissue of the leaves (Figure 1 m, n, o), stems (Figure 1 q, r, s) and roots (Figure 1 u, v, w) and a faint signal detected in the chloroplasts of the leaves and stems. In summary we have detected nuclear staining for SPDS in reproductive and vegetative tissues and some dual cytosol/nucleus localization in the embryo. However, it should be noticed that the limitations of this technique in terms of resolution does not provide unequivocal information with regard to the subcellular localization, therefore we pursued alternative and complementary subcellular localization studies.

### Dual Subcellular Localization of Plant Aminopropyltransferases

**Figure 2:**
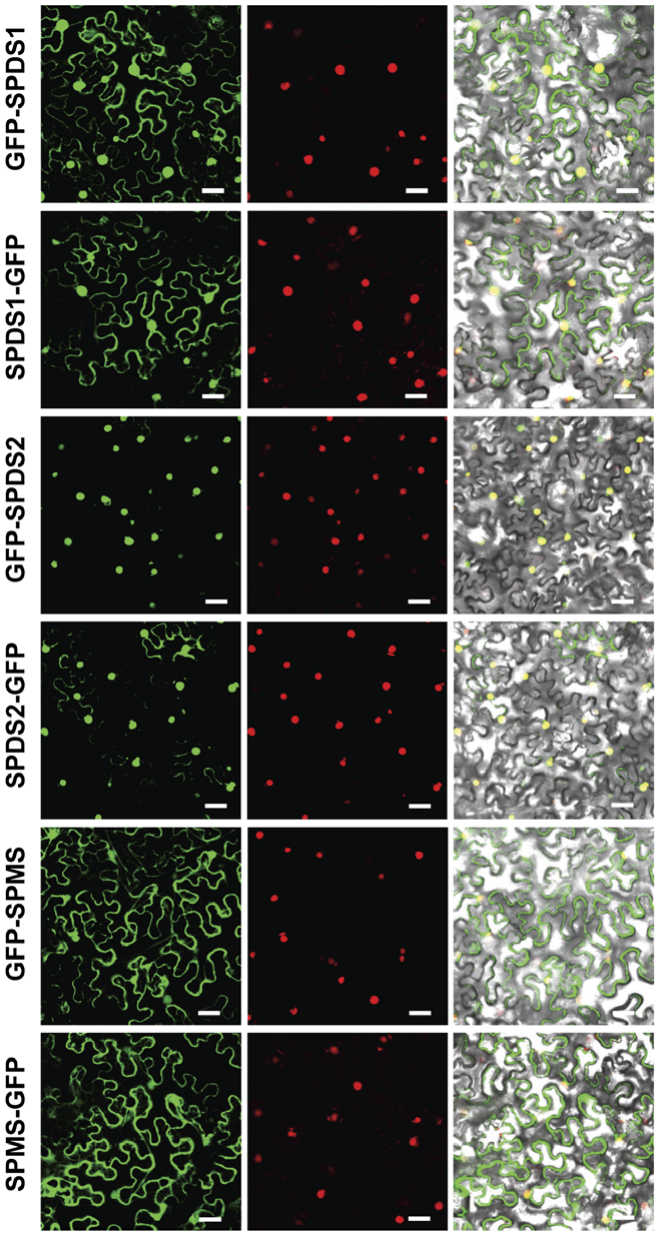
Subcellular localization of aminopropyltransferases as GFP fusion proteins in *N.benthamiana*. Translational fusion constructs of aminopropyltransferases to GFP, both at the N-terminus and the C-terminus were transiently expressed in *N. benthamiana* by agroinfiltration together with a viral nuclear marker fused to mRFP, and analysed with a laser-scanning confocal fluorescence microscope. GFP and mRFP fluorescence spectrum are shown in left and middle column panels. Merged visible and fluoresecent signals are shown in the right column panel. Scale bars: 40 µm.

**Figure 3:**
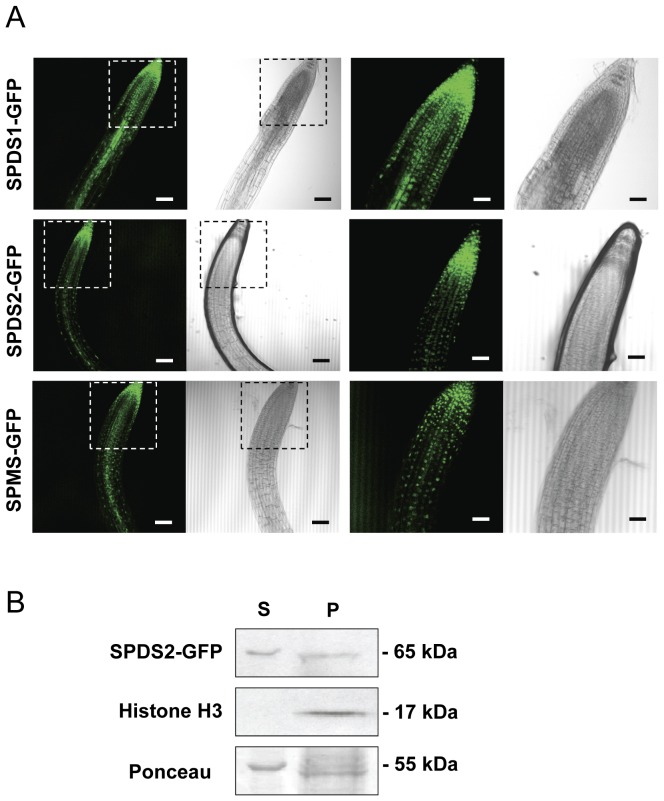
Localization of aminopropyltransferase-GFP fusion proteins and biochemical fractionation of *Arabidopsis* transgenic plants. A, the same constructs in the pMDC83 vector used for transient expression in *N.benthamiana* were used to obtain *Arabidopsis* transgenic plants expressing SPDS1, SPDS2 and SPMS as GFP fusion proteins. T2 transgenic plants were selected and used to visualize the GFP fluorescence or the transmitted signal with the laser-scanning confocal microscope. The insets present part of the same areas with higher magnification. Scale bars: 80 µm for the first two column panels, and 40 µm for the next two column panels. At least 10 different seedlings for each construct were analyzed with similar localization pattern. B, SPDS2-GFP transgenic plants were used for biochemical fractionation of soluble cytosol (S) or nuclear enriched pellet fractions (P) and tested by western blotting using anti-SPDS2 affinity purified antibodies, anti-histone H3 antibodies as a nuclear marker, and Ponceau-S staining of the Rubisco protein as a cytosolic marker.

**Figure 4:**
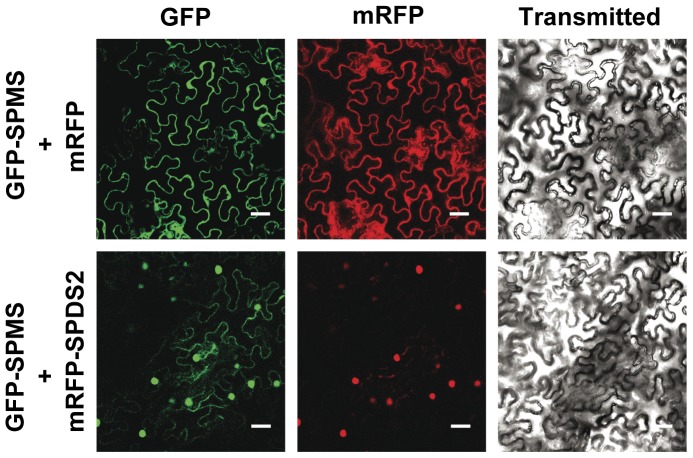
Nuclear localization of SPMS mediated by the presence of SPDS2 in the nucleus of *N.benthamiana*. Agroinfiltration studies were performed in *N.benthamiana* to achieve simultaneous co-expression of GFP-SPMS with either the mRFP fluorescent protein or the translational fusion mRFP-SPDS2 and analysed with a laser-scanning confocal fluorescence microscope. GFP fluorescence spectrum, mRFP fluorescence spectrum, and the visible range are shown for every co-transformation. Scale bars: 40 µm.

As a parallel approach to investigate the subcellular localization of aminopropyltransferases we have used GFP translational fusions to perform both transient expression in *N. benthamiana* by leaf agroinfiltration and stable expression in *Arabidopsis* transgenic plants. We have tested both N-terminal and C-terminal translational fusions to GFP for each of the aminopropyltransferases: SPDS1, SPDS2 and SPMS in *N. benthamiana*. We have excluded TSPMS (*ACL5*) from these studies due to its evolutionary distance and lack of physical interaction with other aminopropyltransferases as previously reported [8]. To assess the fluorescent nuclear signal, every construct was co-infiltrated in *N. benthamiana* together with a red fluorescent nuclear marker containing the nuclear localization signal of the SV40 virus [22]. As shown in Figure 2, while SPDS2 displayed a prominent nuclear localization for both fluorescent constructs, SPDS1 translationally fused to GFP appeared both in the nucleus and the cytoplasm of tobacco epidermal cells, with a more intense nuclear localization when GFP was fused at the N-terminus. SPMS showed a cytoplasmic localization pattern apparently excluded from the nucleus for both constructs. The aminopropyltransferase N-terminal translational fusions to GFP were also used to obtain *Arabidopsis* transgenic plants. *Arabidopsis* transgenic plants were used to evaluate with the confocal microscope the subcellular localization of the aminopropyltransferase fluorescent translational fusions. We concentrated our observations in the root meristem where nuclear localization is clearly visible. As shown in Figure 3A all three aminopropyltransferases displayed a dual nuclear/cytosolic fluorescent signal in the root proximal meristem. Upon approaching the transition zone and more evidently in the elongation and differentiation zone, SPDS-GFP displayed a fluorescent signal restricted to vascular cells, possibly phloematic cells, in agreement with the immunohistochemical data. Similarly, the fluorescent signal of SPMS-GFP displayed a similar pattern to the spermidine synthases. To further investigate the subcellular localization by means of other techniques, we performed biochemical fractionation studies. To this end, we used the SPDS2-GFP transgenic plants for western blot analysis of the cellular fractions (Figure 3B). Although a large portion of SDPS2-GFP was found in the cytosolic crude fraction (Figure 3B lane S), a substantial proportion of the protein was found in the nuclear enriched fraction (Figure 3B lane P). As a nuclear marker we used antibodies against histone H3 showing no cross-reactivity with the cytosolic fraction. The signal of SPDS2-GFP in the nuclear enriched fraction is unlikely to derive from other organelles since the detergent step used for nuclei washing serves to solubilize most proteins from the lysed organelles. It should be noted, however, that this is not a quantitative analysis since it is not possible to recover all nuclei from intact organisms. Therefore, according to the results obtained by means of transient and stable expression with GFP translational fusions, endogenous immunostaining, and biochemical fractionation, a dual cytosol/nuclear localization for both SPDS proteins can be assigned. To reconcile the apparent discrepancy for the subcellular localization of SPMS between the transient heterologous expression in *N. benthamiana* and the ectopic expression in *Arabidopsis*, further experiments were carried out. Taking into account the preferential nuclear localization of SPDS-GFP fusion proteins and the reported physical interaction between aminopropyltransferases [8], we considered the possibility that the SPMS subcellular localization might depend on the presence of SPDS proteins. To verify this, we performed co-agroinfiltration experiments in *N. benthamiana* using the GFP-SPMS construct together with the translational fusion of SPDS2 to the red fluorescent protein mRFP (mRFP-SPDS2). As shown in Figure 4, the presence of SPDS2 leads to a shift in the subcellular localization for SPMS to the nucleus. Therefore, the nuclear localization of SPMS depends on the presence of SPDS proteins in such compartment, and the variable location of SPMS may report the formation of protein complexes in vivo.

### Nuclear Localization of Aminopropyltransferase Enzyme Complexes

**Figure 5:**
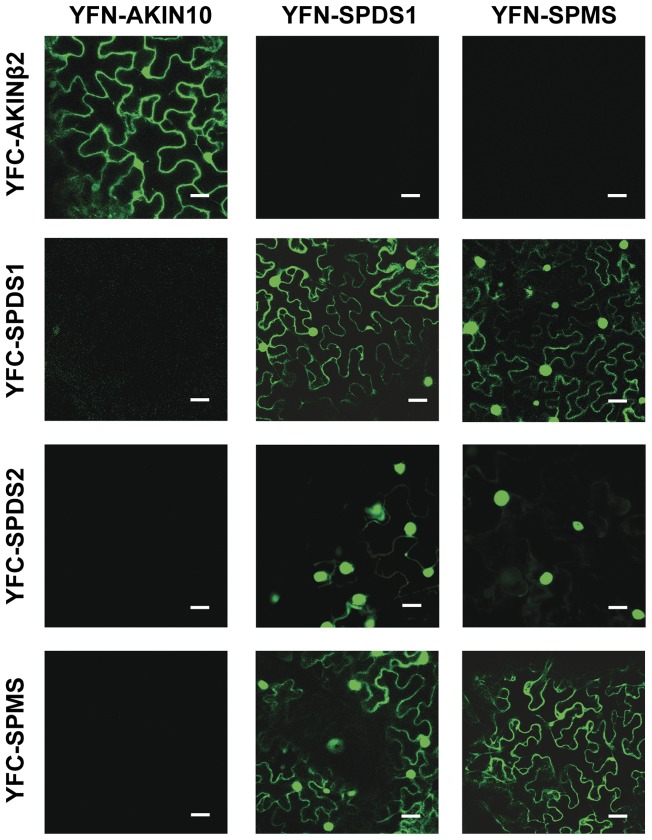
BiFC assays between aminopropyltransferases . Translational fusion constructs of aminopropyltransferase coding sequences to both pYFN43 and pYFC43 were agroinfiltrated into *N. benthamiana* leaves and tested for fluorescence complementation by laser-scanning confocal microscopy. Scale bars: 40 µm.

To determine the subcellular localization of the enzymatic complex formed between aminopropyltransferases previously described [8], we chose the Bimolecular Fluorescence Complementation technique (BiFC) as it allows the non-invasive *in vivo* direct imaging by confocal microscopy of the protein associations under study [14]. The BiFC technique initially established in animal cells was later applied in plants by the development of suitable plant expression vectors [23], [24], however none of those vectors considered the benefit of using recombination-based cloning techniques. We took advantage of gateway-based binary vectors [15] that were adapted for BiFC by constructing the pYFN43 and the pYFC43 binary plasmids (Figure S2). The BiFC vectors were initially tested with positive interaction controls *AKIN10* and *AKINβ2* coding sequences, two subunits of the *Arabidopsis* SnRK kinase [13], showing a clearly visible fluorescence signal under confocal microscope (Figure 5). We then asked whether the aminopropyltransferases SPDS1, SPDS2 and SPMS would show physical proximity within the plant cell and the subcellular localization of those enzymatic complexes. Upon testing for negative controls we detected autofluorescence for SPDS2 constructs in pYFN43, whereas the same construct in pYFC43 gave no background signal. Since possible alterations in the structure of the fusion protein lead to autofluorescence technical problems we omitted any interaction test with SPDS2 in pYFN43. The BiFC-based interaction tests for aminopropyltransferases indicated the assembly of homodimers for SPDS1 and SPMS (not testable for SPDS2 due to autofluorescence) in the same subcellular location as the individual enzymes shown as translational fusions to GFP in Figures 2 and 3A. Heterodimer formation was also apparent for any interaction test evaluated. The most remarkable result was that every heterodimer tested: SPDS1-SPMS, SPDS2-SPMS and SPDS1-SPDS2 occurred mostly within the plant nucleus. Therefore, multienzyme complexes of aminopropyltransferases seem to take place preferentially inside the plant nucleus triggered by the dominant nuclear localization of SPDS proteins.

### Estimation of the Molecular Weight of Aminopropyltransferase Enzymes and Enzyme Complexes

**Figure 6:**
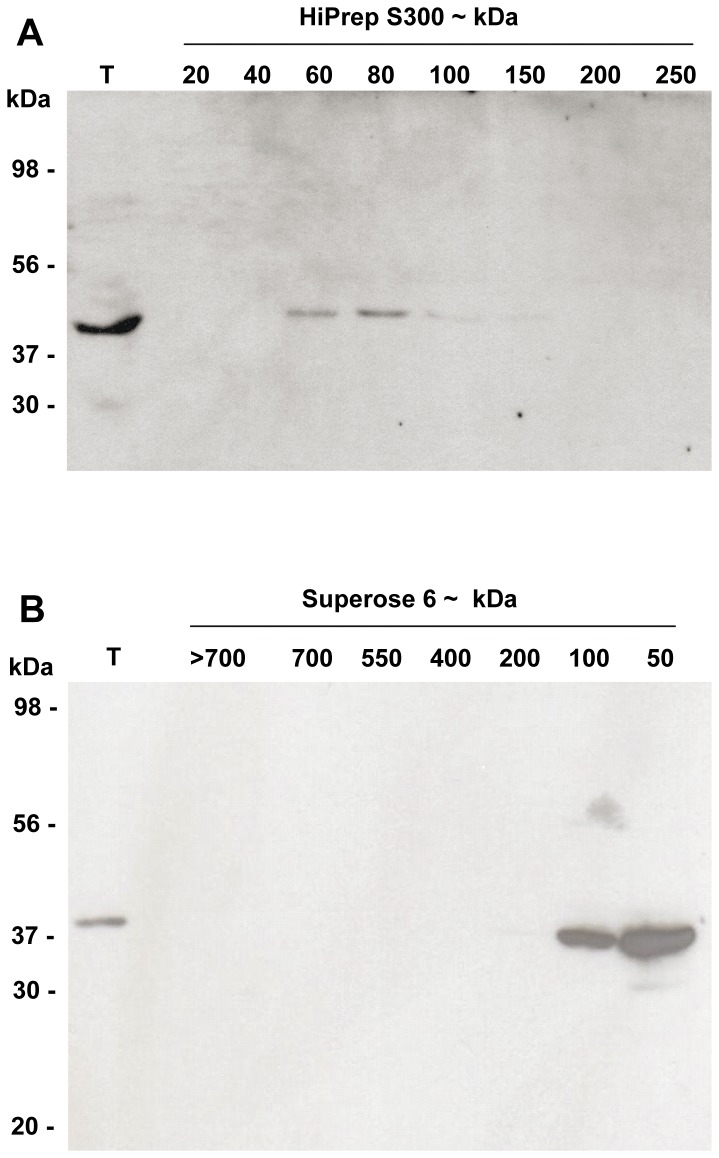
Gel filtration analyses of native SPDS aminopropyltransferases. Total protein extracts from *Arabidopsis* cell suspensions were size fractionated with two different chromatography columns: HiPrep S300 (A) and Superose 6 (B) and tested for immunoblotting with affinity purified anti-SPDS2 antibodies to estimate the apparent molecular size of native SPDS complexes that resulted approximately the size of a dimer.

With the available polyclonal antibody against SPDS we aimed to elucidate the molecular weight of endogenous enzymatic complexes by gel filtration and western blot techniques to compare with previous studies performed with ectopically expressed epitope-tagged versions of the enzymes [8]. Total protein extracts from *Arabidopsis* cell suspensions were size fractionated with two different gel filtration columns: Sephacryl S-300 and Superose 6, and the resulting protein fractions were analysed by western blot against the affinity purified SPDS antibody (Figure 6). The estimated molecular weight for both SPDS1 and SPDS2 is about 37 kDa as it can be seen in the total extract lane for both immunoblots. Both fractionations yielded identical size exclusion pattern for SPDS with the most intense signal corresponding approximately to the size of a protein dimer (about 70 to 80 kDa) and decreased signal at higher molecular weight until 150 kDa. We could not detect SPDS protein complexes of higher molecular weight as previously reported for the ectopic overexpression of epitope-tagged enzymes in *Arabidopsis* cell suspension cultures. The reason for this discrepancy is explained later in the Discussion section.

**Figure 7:**
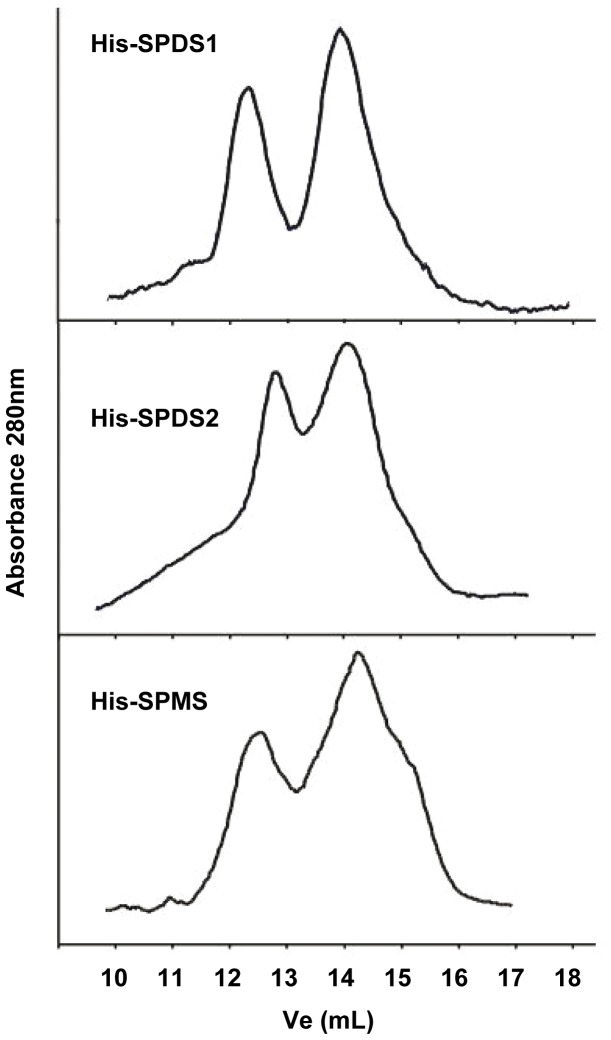
Recombinant aminopropyltransferases can associate as homodimers. Aminopropyltransferase proteins expressed in *E. coli* as fusion proteins to His-tag were affinity purified with nickel chromatography and size fractionated with a Superdex 200 10/300 GL column. 1.5 mL volume fractions were monitored for absorbance at 280 nm. Two peaks corresponding to the size of monomer and dimer were detectable in every case. The gel filtration chromatography was repeated at least twice for each purified recombinant protein and figure shows a representative elution profile.

Since the BiFC data suggested the building of both homodimers and heterodimers for aminopropyltransferases, and our immunoblot data of gel filtration fractions did not allow the discrimination between SPDS homo and heterodimers, we purified His-tagged recombinant enzymes from *E. coli* and performed size exclusion chromatography with the purified enzymes to evaluate the capacity of aminopropyltransferases to assemble as homodimers. The recombinant enzymes purified by nickel affinity chromatography were tested for immunostaining with anti-SPDS and anti-His antibodies (Figure S1, panel B) and subjected to gel filtration chromatography with Superdex 200 column. The protein fractions eluted were followed by absorbance at 280 nm and the elution profile is shown in Figure 7. Two peaks corresponding to the size of the monomer and the dimer can be detected in all cases. Therefore the three *Arabidopsis* aminopropyltransferase enzymes tested: SPDS1, SPDS2 and SPMS show the capability to assemble as homodimers, thus confirming the BiFC data obtained.

## Discussion

The advances in plant molecular biology and plant genetics have facilitated the molecular characterization of plant polyamine biosynthesis pathway, however, in spite of vast information with regard to plant aminopropyltransferase biochemical features, no details related to the subcellular localization of the enzymes and enzymatic complexes can be found in the literature. To address this issue we have used polyclonal antibodies generated against the SPDS2 protein, which do partially cross-react with SPDS1 but do not show any cross-reactivity with SPMS. The immunohistological data from *Arabidopsis* wild type indicate dual cytosol/nucleus localization for SPDS proteins in most of the tissues with detectable signal. We have also used aminopropyltransferase translational fusions to GFP for both transient expression in *N.benthamiana*, and stable *Arabidopsis* transgenic plants. The results showed a similar dual localization for SPDS1 and SPDS2 with a prominent nuclear localization in the case of SPDS2, both in *N.benthamiana* and *Arabidopsis*. However a partial discrepancy was detected in the case of SPMS, since it was excluded from the nucleus after the leaf transient expression in *N.benthamiana*, but displayed some unequivocal fluorescent nuclear signal in transgenic *Arabidopsis* root proximal meristems. A closer look at the root elongation and differentiation zone showed diffused fluorescent signal out of the nucleus, suggesting that SPMS localization might vary depending on the cellular context, perhaps depending on the presence of other proteins. In relation to this, since we had previously reported the physical interaction between all three aminopropyltransferases SPDS1, SDPS2 and SPMS, we hypothesized and confirmed (Figure 4) that the nuclear localization of SPMS could be determined by the presence of SPDS proteins that display a preferential nuclear localization. The reason why SPMS translationally fused to GFP is excluded from the nucleus upon transient expression in *N. benthamiana* may be due to the absence of SPDS orthologous proteins in the tobacco leaf epidermis or to the lack of interaction between *Arabidopsis* and *N. benthamiana* aminopropyltransferases, or both. It is remarkable that no obvious nuclear localization signal could be found in the primary structure for SPDS1 or SPDS2 with sequence analysis programs, so at this point we cannot postulate whether there are cryptic primary or most probably tertiary/quaternary structure determinants for SPDS nuclear localization that could also explain the enhanced nuclear accumulation for SPDS2 compared to SPDS1. Coincident with our observations for plant aminopropyltransferases, a similar nuclear localization pattern has been shown in *Saccharomyces cerevisiae* [25]. Moreover, also similar protein interaction capabilities between the enzymes SPE3 and SPE4 have been described in the baker’s yeast [26]. To further support our observations in plant cells, polyamines have been localized in the nucleus of animal cells [27]. The same authors have documented the appearance of polyamine vesicles as a hypothetical mechanism to remove the polyamine pool from the nucleus and possibly also from the cell.

The subcellular localization data suggested that building of enzymatic complexes could determine the location of polyamine biosynthetic enzymes therefore we aimed to elucidate the subcellular emplacement of the aminopropyltransferase complexes. To address this issue we developed gateway-based binary vectors for BiFC named pYFN43 and pYFC43 (Figure S2). The results of the BiFC assays with aminopropyltransferases showed a predominant nuclear localization for aminopropyltransferase heterodimers (Figure 5). The BiFC analysis also showed the capability to form homodimers for all tested aminopropyltransferases (SPSD1, SPDS2 and SPMS). To further validate our positive data of homodimer assembly by BiFC, we analyzed by gel filtration the behaviour of recombinant purified enzymes showing their capability to form homodimers *in vitro* (Figure 7). In agreement with our results, to date all characterized aminopropyltransferases are homodimers [28], [29], [30], [31], [32] with the only exception of acute thermophiles, where aminopropyltransferases display a tetramer structure formed by pairs of homodimers [33]. Detailed structural analysis of aminopropyltransferase enzymes bound to substrates, products, and inhibitors have provided valuable information with regard to structural dimerization requirements that involve either the N-terminal region for human SPMS [32] or both the N-terminal and the C-terminal domains for the human SPDS enzyme [31]. In the course of this investigation we have found discrepancies to the previous description of the estimated size of the multienzymatic protein complexes. Our current studies with endogenous protein complexes rather indicate that aminopropyltransferases behave as dimers *in vivo* but do not take part in multiprotein complexes of larger size as estimated previously [8]. Such overestimation could have occurred due to interferences with immunoglobulins that might have artificially increased the estimated size of immunopurified enzyme complexes.

The data presented here do not reveal which type of aminopropyltransferase protein dimer is more frequently occurring in the cell, either the homo or the heterodimer. In any case, it seems that spermine biosynthesis enzymes can assemble as heterodimers with spermidine synthases inside the nucleus. To our knowledge, this is the first example of aminopropyltransferase enzyme complexes taking place inside the nucleus. However, we should remark that such protein interaction between SPDS and SPMS might not reveal an enzymatic channel for the substrate spermidine, but it could also represent an alternative enzymatic structure with regulatory functions as it has been described in the case of the cysteine synthase complex [34]. Why spermidine as a substrate seems to require metabolon-like structure assembly within the nucleus and how it is regulated remains to be solved. But also why spermidine and spermine biosynthesis in plants seem to take place inside the nucleus is an open question arising from this work that demands future research efforts.

## Supporting Information

Figure S1 **Immunoblot detection of aminopropyltransferase proteins.** Panel A shows the detection by western blot of SPDS proteins (indicated by asterisk) from a total plant protein extract after SDS-PAGE separation and blotting using either affinity purified antiserum against SPDS2 protein (left panel) or crude serum against GST-SPDS2 fusion protein (right panel). Panel B shows the purification of recombinant aminopropyltransferases fused to His-tag and immunological detection of purified proteins with either anti-SPDS2 crude serum antibody or monoclonal anti-His antibody.
(TIF)

Figure S2 **Design of gateway-based BiFC binary vectors pYFN43 and pYFC43.** The figure shows the schematic diagram of the T-DNA fragment of both pYFN43 (A) and pYFC43 (B) plant expression binary vectors that allow gateway-based LR recombination for BiFC assays.
(TIF)
