# Supplementary figures and images for: Aminopropyltransferases Involved in Polyamine Biosynthesis Localize Preferentially in the Nucleus of Plant Cells
Source: PLoS One. 2012 Oct 8;7(10):e46907. doi: 10.1371/journal.pone.0046907 (PMC3466176; doi:10.1371/journal.pone.0046907)

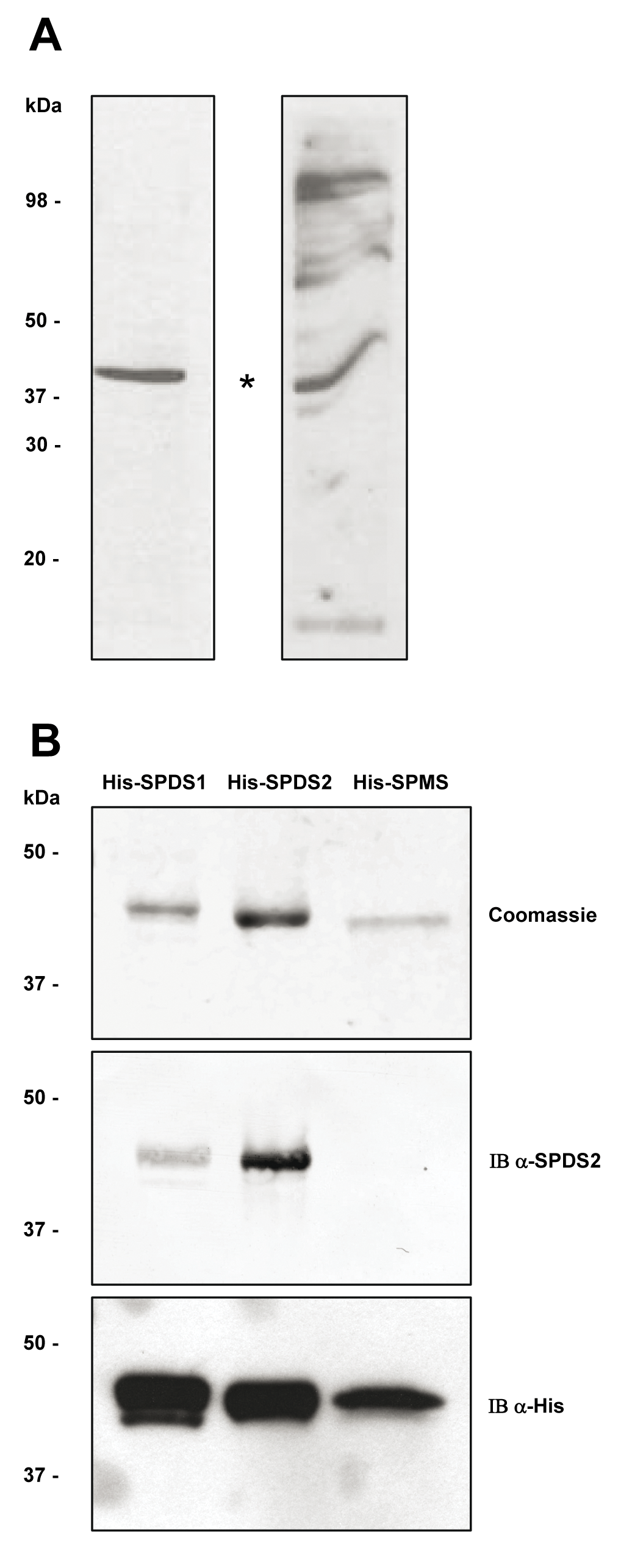

Supplement: Figure S1 — Immunoblot detection of aminopropyltransferase proteins. Panel A shows the detection by western blot of SPDS proteins (indicated by asterisk) from a total plant protein extract after SDS-PAGE separation and blotting using either affinity purified antiserum against SPDS2 protein (left panel) or crude serum against GST-SPDS2 fusion protein (right panel). Panel B shows the purification of recombinant aminopropyltransferases fused to His-tag and immunological detection of purified proteins with either anti-SPDS2 crude serum antibody or monoclonal anti-His antibody. (TIF) [file pone.0046907.s001.tif]

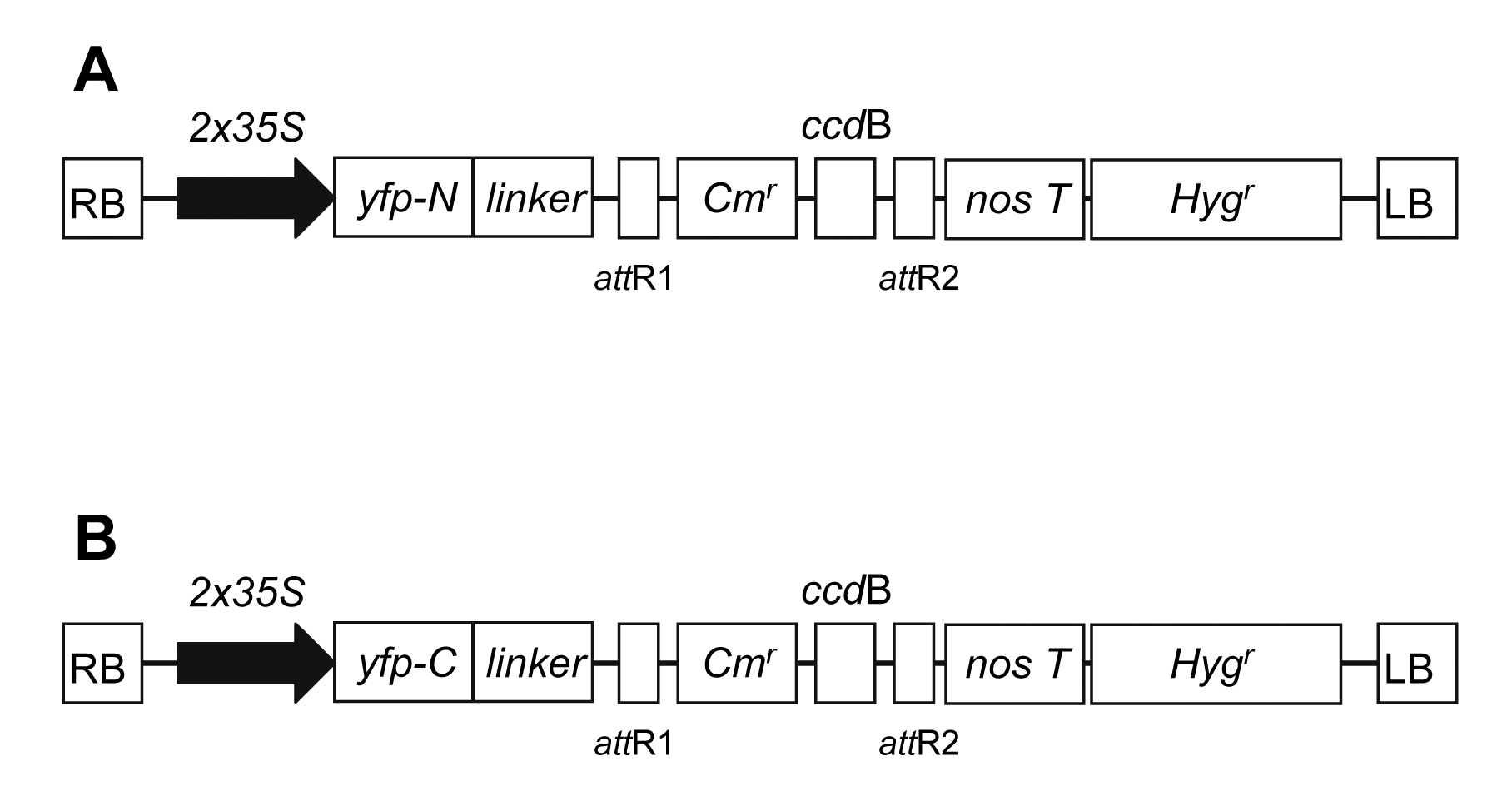

Supplement: Figure S2 — Design of gateway-based BiFC binary vectors pYFN43 and pYFC43. The figure shows the schematic diagram of the T-DNA fragment of both pYFN43 (A) and pYFC43 (B) plant expression binary vectors that allow gateway-based LR recombination for BiFC assays. (TIF) [file pone.0046907.s002.tif]
